# Deliberate Foreign Body Ingestion in a 35-Year-Old Woman With Borderline Personality Disorder and Several Psychiatric Comorbidities

**DOI:** 10.7759/cureus.13179

**Published:** 2021-02-06

**Authors:** Fariha Bangash, James L Megna, Luba Leontieva

**Affiliations:** 1 Psychiatry, State University of New York Upstate Medical University, Syracuse, USA; 2 Psychiatry and Behavioral Sciences, State University of New York Upstate Medical University, Syracuse, USA; 3 Psychiatry, State University of New York Upstate Medical University, Syracuse, USA

**Keywords:** borderline personality disorder, deliberate foreign body ingestion, post-traumatic stress disorder, childhood sexual abuse, repeated self-injurious behavior

## Abstract

Deliberate foreign body ingestion (DFBI) is a rare psychopathological disorder that involves the swallowing of non-nutritive objects to cause self-harm. It is most commonly associated with borderline personality disorder (BPD). Very scant literature has been published on the psychopathological understanding or psychopharmacological interventions. Mostly, gastroenterological and surgical management regarding the removal of the foreign body has been discussed in the literature. DFBI can be very challenging in terms of the treatment of the patient and the morale of the health providers - it exhausts the patient and the family and evokes frustration among the medical staff due to its resistance to remission. By presenting the case of a patient in this article, we will discuss what is known about the poorly understood DFBI and the challenges and difficulties encountered while treating these patients. Further, we will discuss how a biopsychosocial approach can be used in treating these patients.

## Introduction

Deliberate foreign body ingestion (DFBI) is a form of self-harm behavior that involves swallowing non-nutritive objects. The presence of foreign bodies in the intestines of patients was first described by Baudamont in 1779 [1]. DFBI has been seen in the following patient populations: malingering, psychosis, pica, and personality disorders [2]. In a study of 292 foreign-body ingestion cases by Palta et al., 92% of all cases were deliberate, 85% were psychiatric patients, and 84% of cases were individuals with previous ingestions [3].

Repeated DFBI is mainly associated with borderline personality disorder (BPD) [4]. The Diagnostic and Statistical Manual of Mental Disorders, 5th Edition (DSM-5) defines borderline personality disorder as “a pervasive pattern of instability of interpersonal relationships, self-image, and affects" [5]. A diagnosis of BPD requires the presence of at least five of the following criteria; efforts to avoid real or perceived abandonment; unstable interpersonal relationships; persistent, unstable self-image; marked impulsivity; recurrent suicidal or self-mutilating behavior; unstable affect or mood; chronic feelings of emptiness; difficulty controlling anger; and transient paranoia or dissociative symptoms. The lifetime prevalence of BPD is 0.5%-5.9%. Self-harming behaviors are very common in BPD patients; around 80% of BPD patients will self-harm at some point, and over two-thirds make suicide attempts [4].

Based on a comprehensive review published in 2014, there were only 15 case reports to date on DFBI patients with personality disorders. Of these cases, most of the patients were younger female patients with BPD diagnoses who also had long histories of self-harm behaviors and had multiple prior hospitalizations [6]. In personality disorders, the patients use intentional foreign body ingestion as a method of self-harm [2]. The case presented here is an example of repeated foreign body ingestion as a form of self-harm in a patient with BPD and its challenges for the amelioration of psychopathology.

Treatment of DFBI also requires economic consideration, as patients are seen by multiple specialties, including the emergency department (ED), anesthesia, surgery, psychiatry, nursing, and even security services. Around 1500 deaths are attributed to foreign body ingestions per year in the US alone [7], 92% of which are deliberate. The average cost of a single patient per hospital visit is $6616 in the US and $2305 in Canada [7]. Huang et al. report that the total cost for 33 patients being treated for DFBI across a total of 305 hospital visits in an eight-year period was just over $2,000,000 [8]. The average inpatient admission duration of stay was 5.66 days.

With regards to the treatment of DFBI, emphasis has been primarily given to surgical and gastroenterological management. This is because DFBI often accompanies serious physical complications such as esophageal and/or bowel obstruction, perforation, and hemorrhage [1]. However, there is little understanding regarding the underlying psychopathology and psychiatric management of the behavior. This leads to poor understanding of the behavior as well as limited psychotherapeutic management for patients. There is some evidence that a biopsychosocial approach can be used in treating these patients to develop an individualized treatment plan to prevent the repetitive behavior [9]. This would require a multidisciplinary approach, including pharmacological and psychological therapies.

The goal of this article is to address the challenges encountered while treating these patients and to discuss a management plan and underlying features of this behavior by presenting the case of a young woman who was diagnosed with borderline personality disorder and used repeated DFBI as a form of self-harming behavior.

## Case presentation

The patient is a 35-year-old Caucasian woman of north American origin with a 20-year history of repeated, deliberate ingestion of foreign bodies. Over the years, the objects she had ingested included batteries, razor blades, pen caps, screws, roofing nails, and reading glasses. The most frequently ingested objects were razor blades. The patient has undergone multiple endoscopies, surgeries, admissions in psychiatric wards, and long-term hospitalizations in psychiatric institutions. Additionally, she would swallow foreign bodies during her stay in psychiatric institutions. The patient continued to be in and out of foster homes, hospitals, long-term psychiatric units, and community residences for repeated swallowing of foreign bodies such as pen caps, screws, and batteries.

The patient was born on a farm to a mother with alcohol abuse disorder. She was the second of five siblings, one of whom was stillborn with fetal alcohol syndrome. Her mother drank and smoked heavily while pregnant with the patient. The patient was verbally and emotionally abused by her mother and experienced feelings of rejection and abandonment from a very young age. The only support and close relationship in her family were with her father. She recalls herself as being “daddy’s little princess.” Her father was her comfort whenever she felt rejected, unwanted, or abused. When she was four years old, the patient was sexually assaulted by her 13-year-old male cousin, and her father was the one who had found them and removed him from her. She was taken to the doctor two days later for a check-up because her mother found her bleeding vaginally.

The patient witnessed her father’s death at the age of nine when he was accidentally crushed by his own car while repairing it. She says that her mother was inside the house getting intoxicated at the time and thus she blames her mother for his death. After her father’s death, the patient’s mother remarried. The patient was sexually abused by her step-father and 38-year-old step-brother from the age of nine to fifteen. Her mother gave consent to this situation and allowed it to happen for years until the patient moved away to live with her grandmother. By this time, the patient began engaging in self-injurious behaviors (i.e. cutting her wrists). The patient attempted to jump off of a bridge and was admitted into a psychiatric unit for the first time. Upon discharge, she went through a series of foster care homes and was admitted to various hospitals for attempted suicide. The patient started swallowing non-nutritive objects and fluids when she was 15. Her first attempts involved swallowing batteries, as well as drinking out of perfume and inhaler bottles at her foster home.

The patient continued to be in and out of foster homes, hospitals, long-term psychiatric units, and community residences through the age of 18 for repeated swallowing of foreign bodies such as pen caps, screws, and batteries. At age 18, she was emancipated from the state and allowed to live independently. She has been arrested twice on charges of criminal mischief and spent several days in jail for the charges. The patient has not had a stable home for the past 20 years and has been unable to maintain a long-term job.

The patient lacks any stable, nurturing relationships. As an adult, she was allowed to see her mother at her home on weekly visitations. She describes these visits as cold, with her mother being intoxicated and indifferent to her presence. Her mother passed away from lung cancer in 2014. She mourns the loss of her mother despite their estranged relationship. The patient had unstable romantic relationships throughout her adult life. She was in two long-term relationships that were verbally and physically abusive. At one point, she suffered stress-induced seizures and transient ischemic attacks because of the abuse. The patient had a miscarriage in August 2012 and lost a baby boy. This was another emotionally stressful event in her life, and she started swallowing objects again immediately after. Over the next four years, she swallowed razor blades on multiple occasions.

The patient started drinking at age nine after her father passed away, and she struggled with alcohol dependence for many years. She attempted sobriety a few times. She has currently been sober for the past 12 years. The patient became morbidly obese over the years and decided not to swallow for two years so that she could undergo gastric sleeve surgery in 2018. She was successful in losing 324 pounds over the next two years. She has undergone multiple surgeries for gastric perforations after swallowing razor blades.

The patient has been admitted into a long-term state psychiatric hospital six times. All her hospitalizations were related to escalations of swallowing objects. At the age of 19, her hospitalization lasted nine months. She was diagnosed with adjustment disorder with depression and borderline personality disorder. She was discharged on bupropion XL, carbamazepine, naltrexone, and lamotrigine. The records indicated that the addition of naltrexone played a key role in curtailing her impulsivity and secondary gain from getting attention after swallowing and allowed her to participate in psychotherapy.

At age 25, she had another state psychiatric hospital admission for one month, again due to foreign body ingestion. At that time, she was described as “detached from emotions.” She received dialectical behavior therapy and hypnosis for the treatment of her flashbacks and anxiety. Her diagnoses remained adjustment disorder with depression and borderline personality disorder. During that admission, she complained of hearing voices, so she was prescribed haloperidol, lithium for impulsivity, quetiapine for anxiety, zolpidem for sleep, and benztropine for antipsychotic side effects correction.

Her most recent admission to the hospital was in August 2020. This was around the time of her son’s death anniversary. The patient had swallowed four roofing nails that passed through the gastrointestinal tract. Upon her discharge from the psychiatric unit, she presented back to the ED within two hours after swallowing 10 razor blades. The patient has been under treatment in the psychiatric ward till date. Her current diagnoses are BPD, post-traumatic stress disorder, unspecified psychotic, depressive, and anxiety disorders. She is being treated with clonidine, lurasidone, escitalopram, venlafaxine, prazosin, tizanidine, zolpidem, and alprazolam, which was later replaced by clonazepam for reactive impulsivity issues. In addition, she is receiving psychotherapy treatments aimed at emotion regulation, problem-solving, and functional assessment. These include daily dialectical behavior therapy (DBT), daily group therapy, and individual psychotherapy sessions.

As the death anniversary of her father was approaching closer, the patient was becoming more depressed. She started experiencing flashbacks and having dreams about her father that made her more anxious, irritable, dysphoric, secluded, and hypervigilant. She became aggressive toward the nursing staff - being both verbally abusive by calling them names and physically abusive by throwing empty plastic bottles at them. She told her caregivers that she was having urges to swallow her reading glasses. Her concerns were addressed, and she was given individual therapy sessions to handle the stress and impulsiveness. Eventually, however, she did swallow the side piece of her reading glasses.

## Discussion

Self-harm behaviors have been associated with patients diagnosed with severe personality disorders, post-traumatic stress disorder (PTSD), and some psychotic disorders [2]. Deliberate self-harm, parasuicide, repeated self-injury are synonymous terms that have been used to describe a patient’s self-harming behavior. Self-injurious behavior has often been associated with a history of abandonment and childhood physical and/or sexual abuse [2]. Additionally, a history of childhood physical/sexual abuse in conjunction with PTSD has been associated with BPD and self-harming behaviors [10].

In patients who present with repeated behaviors of self-harm, the intent is usually non-suicidal or para-suicidal [2]. This non-suicidal form of self-harm is used as a means to survive difficult situations in which one has little or no control. These patients are also generally filled with anxiety, irritability, and anger. They can be overtaken by sudden, recurrent impulses to harm themselves and usually do not resist these urges. [4]

Our patient, who was diagnosed with BPD and PTSD, had suffered childhood sexual abuse for several years before the age of 15. The patient had internalized feelings of shame and guilt and wanted to punish herself for being a part of those sexual acts. As a result, she started cutting herself at the age of 15 with progression to foreign body ingestion as a form of self-punishment. She believes she performed those “shameful and horrible sexual acts” of her own volition. She has a poor understanding of the fact that she was a minor at the time and couldn’t defend herself. The patient attempts to find relief from these feelings through suicidal ideations and self-harming behaviors, including the ingestion of foreign bodies. She often acts without considering the consequences of her actions. The act of self-harm serves to overcome the extreme painful turmoil that goes inside her.

Gregory et al. suggest a theory about the motivation for self-injury in adolescents. According to this theory, which he refers to as “magical thinking,” self-harm behavior is used as a means of relieving negative emotions or stress when individuals are unable to adequately cope [11]. Magical thinking was first defined by Piaget (1962) as the “pre-symbolic use of language that lacks differentiation between the real and the symbolic or between signifier and signified.” During puberty, adolescents undergo hormonal changes that are responsible for structural changes in parts of the brain, including the prefrontal cortex and limbic areas that are involved in the regulation and processing of emotions. The resulting functional changes hinder the brain from effectively translating emotions into language [11]. This explains why these individuals have limited capability for verbalizing emotions. There is an established link between magical thinking and BPD [12].

Non-suicidal self-harming behaviors may have an underlying factor of seeking help or communicating with others [13]. Our patient’s attempts at repeated self-harm have often been aimed at seeking attention from and gaining control over her care-givers. The first time she ingested foreign bodies was when she was living in a foster home. She swallowed batteries and immediately after, told the caregiver who took her to the hospital. Our patient engages in self-harming behaviors specifically around the time of the death anniversary of a loved one. The swallowing episode is usually preceded by mounting tension, increased agitation, uncontrollable anxiety, and depression culminating in the act of self-harm. The self-injurious behavior serves as a means of providing relief from these affective states.

Taken together, a theoretical model to understand the patient’s deliberate foreign body ingestion would include the defense mechanism of incorporation in an effort to diminish extreme anxiety associated with feelings of abandonment and emptiness. This, of course, began as a totally unconscious behavior, but with repetitions over many years, reached a level of conscious awareness that allowed the patient more control over anxiety reduction. This was reinforced by staff responsiveness to her needs (secondary gain) as a result of clinical/medical problems produced by her ingestions.

A biopsychosocial formulation form created by the psychiatric in-patient unit at the patient’s hospital was applied to the patient. This formulation utilizes a multimodal approach to understand the patient’s underlying psychopathology by taking into consideration biological, psychological, and social factors surrounding the patient.

It was assessed that from a biological perspective, the patient’s current diagnoses have been a result of her history of childhood sexual abuse. Given the history of her mother’s alcohol dependence disorder, this may have had effects on the patient’s developing brain. Previous research suggests that individuals with a history of fetal alcohol syndrome or fetal alcohol effects experienced psychiatric and developmental disorders and needed psychiatric treatment later in life [14]. The patient herself also has a history of alcohol dependence disorder from a very young age that she reports has been resolved. The comorbidity of BPD and substance use disorder, particularly alcohol abuse, has been documented in research [15].

From a psychological perspective, the level of personality organization is borderline/immature. Borderline defenses include projective identification, splitting, over-idealization/devaluation, and immature defenses include acting out, somatization, and turning against self. Combined, these contribute to mood lability, impulsivity, and hostility. Finally, from a social and developmental perspective, the patient’s early experiences were characterized by neglect and emotional abuse from her mother. During later years, she suffered physical abuse from her romantic partners. Consequently, an interpersonal pattern of hostile dependency developed.

Treatment for DFBI patients should be characterized by “compassion, dignity, and respect” [16]. Counter-transference reactions from medical staff can be evoked while dealing with the patients of DFBI. Medical and hospital staff may become frustrated by repeated admissions of such patients. When staff act on their countertransference, however, the patient may feel encouraged to repeat the same behaviors in the future as a result of defensive acting out. A suggested solution is to hold multidisciplinary meetings in which staff counter-transference reactions can be addressed without negatively affecting patient care. A professional and neutral approach by medical staff will reduce the likelihood that patients feel blamed or judged for their actions [16].

Pharmacological treatment with naltrexone and clonidine has been shown to decrease the impulsivity and frequency of self-harm [17]. The endogenous opioid system has been suggested to be involved in the pathological development of self-harm behavior. Long-acting opiate antagonists, such as injectable naltrexone, are effective in the reduction of recurrent self-injurious behavior [17]. In female patients with BPD, clonidine has shown promising results in alleviating symptoms of dissociation, aversive inner tension, and self-harming impulses [18].

Other proposed psychotropic medications used for the treatment of BPD include:

1. Serotonergic and combined serotonin-norepinephrine reuptake inhibitors (SSRIs, SNRIs) for anxiety, depression, and impulsivity; there is an interaction between the 5-HTTLPR polymorphism and early life events, which may lead to the development of impulsivity in patients with BPD [4];

2. Benzodiazepines are used for excessive anxiety and irritability on an as-needed basis primarily and only for time-limited periods; special care should be taken in treatment with short-acting benzodiazepines, as they may cause impulsive reactivity in patients with BPD who are already predisposed to impulsivity [19];

3. Mood stabilizers, including lithium and anti-epileptic agents for mood lability and impulsivity [9];

4. Antipsychotics for controlling aggression [9];

5. Monoamine oxidase (MAO) inhibitors and tricyclic antidepressants for depression [9];

6. Beta-blocking agents for hyperarousal, dissociation, and impulsiveness [9].

Studies have shown dialectical behavior therapy to be the most promising treatment for individuals with self-injurious behavior [20]. The American Psychological Association suggests outpatient DBT lasting more than one year for patients with a borderline personality disorder [9]. The three main treatment goals of DBT are: stability and security; reduction of post-traumatic stress; and a sense of achievement of self-respect and life goals [20]. DBT has been shown in many studies to decrease self-injury, hopelessness, and depression in these patients [2].

Dynamic deconstructive psychotherapy (DDP) has been developed for more challenging patients with BPD with co-occurring substance abuse, reckless behaviors, and parasuicidal behaviors [12]. Patients with BPD are hypothesized to have impairments in neurocognitive functioning due to atrophic changes in the hippocampus, amygdala, anterior cingulate gyrus, and prefrontal cortex. The specific neurocognitive deficits are characterized by the inability to accurately identify, associate, and understand different experiences they may have had. DDP aims to help in establishing and utilizing these neurocognitive functions so that the patient may have a better sense of self-awareness. The therapy generally lasts for a period of 12-18 months [12].

Prevention and treatment of DFBI in patients with BPD and PTSD require a multidisciplinary approach including psychopharmacological interventions and psychological therapies. Specific prevention strategies during inpatient admission include continual observation of patients, removal of potential ingestible objects, a care plan aimed at preventing reinforcement of behavior, and treatment with medications and psychotherapy.

## Conclusions

DFBI is a difficult-to-treat disorder that is frequently seen in patients with personality disorders, particularly BPD. Treatment of DFBI requires a multidisciplinary approach, with a combination of psychological therapies and psychopharmacologic interventions. DBT and DDP have promising results as psychological treatment options. Psychopharmacology would include naltrexone, clonidine, SSRIs, SNRIs, a short-term course of as-needed benzodiazepines, mood stabilizers, neuroleptics, MAO inhibitors, tricyclic antidepressants, and beta-blocking agents.
